# The Gene Flow Direction of Geographically Distinct *Phytophthora infestans* Populations in China Corresponds With the Route of Seed Potato Exchange

**DOI:** 10.3389/fmicb.2020.01077

**Published:** 2020-05-26

**Authors:** Fangluan Gao, Changsheng Chen, Benjin Li, Qiyong Weng, Qinghe Chen

**Affiliations:** ^1^State Key Laboratory of Ecological Pest Control for Fujian and Taiwan Crops, Fujian Agriculture and Forestry University, Fuzhou, China; ^2^Fujian Key Laboratory for Monitoring and Integrated Management of Crop Pests, Institute of Plant Protection, Fujian Academy of Agricultural Sciences, Fuzhou, China

**Keywords:** *Phytophthora infestans*, mitochondrial DNA, genetic diversity, population structure, migration pattern

## Abstract

*Phytophthora infestans* is a widespread destructive plant pathogen that causes economic losses worldwide to potato production. In this study, we sequenced four mitochondrial DNA gene sequences of 101 *P*. *infestans* isolates from five potato-growing regions in China to investigate the population structure and dispersal pattern of this pathogen. The concatenated mtDNA sequences in the populations showed high haplotype diversity, but low nucleotide diversity. Although there was a degree of spatial structure, our phylogeographic analyses support frequent gene flow between populations and the direction of gene flow, primarily from north to south, corresponds to the route of seed potato transportation, suggesting a role of human activities in the dispersal of *P*. *infestans* in China.

## Introduction

Gene flow, or migration, refers to the movement of alleles among spatially or temporally separated populations and is a major driving force in the evolution of populations. Gene flow may increase the genetic variation of local pathogen populations but decrease the genetic difference between populations, which can slow or limit population differentiation (Endler, 1973; Zhan, 2016). Population genetics studies have contributed to our understanding of the evolutionary history of plant pathogens, as well as to the development of strategies to prevent their spread in major crops (Mcdonald and Linde, 2002). For example, some soil-borne or seedborne plant pathogens, such as the fungal *Cryphonectria parasitica*, are spatially structured and quarantine procedures can be effective in preventing the invasion of plant diseases caused by the pathogens (Gilbert, 2002; Horton, 2010).

*Phytophthora infestans*, the causal agent of the Great Irish Famine, is a highly destructive plant pathogen, causing worldwide losses in potato production of $6.7 billion annually (Haverkort et al., 2008). Knowledge of population genetics and the evolutionary biology of *P*. *infestans* are important for developing sustainable, effective strategies for controlling potato late blight. In population genetics, selectively neutral loci, such as microsatellites (simple sequence repeats, SSRs), are the most frequently used genetic markers (Kirk and Freeland, 2011) and have been employed to investigate the evolutionary dynamics and population structure of *P*. *infestans* (Goodwin, 1997; Lebreton et al., 1998; Li et al., 2012). However, SSRs are often expensive and time-consuming (Vieira et al., 2016). Mitochondrial DNA (mtDNA) is now a popular marker because of its utility in investigating evolutionary processes, such as migration or dispersal (Holderegger et al., 2006; Wang et al., 2020). Cardenas et al. (2011) conducted a population genetics analysis of *P*. *infestans* in Colombia and Venezuela using four nuclear (*ITS*, *Ras*, *β-tubulin*, and *Avr3a*) and one mitochondrial (*Cox1*) gene, and revealed the low genetic diversity and low frequency of heterozygotes in *P*. *infestans*.

Potato (*Solanum tuberosum* L.) is the fourth most important staple food crop worldwide. China is the world’s largest producer of potatoes, producing ∼99.21 million tonnes on ∼5.76 million ha in 2017^1^. With government support and shifts in dietary habits, potato production in China is expected to increase substantially in the coming decades (Kearney, 2010). Potato planting in China can be roughly divided into four producing regions according to climate conditions and management practices: the northern single-cropping, central double-cropping, winter-cropping, and southern mixed-cropping zones (Jansky et al., 2009). Of these, the northern single-cropping zone, including Heilongjiang Province, is the major producer of potatoes and accounts for 50% of China’s total potato yield.

The existence of *P. infestans* in Kunming and Chongqing of China was noted in as early as 1938 and 1940, respectively (Ristaino and Hu, 2009). Molecular population genetics approaches have been used to investigate the genetic diversity of *P*. *infestans* in China, and to identify the evolutionary forces responsible for its diversity. For example, based on SSRs and amplified fragment length polymorphisms (AFLPs), Guo et al. (2009) found low genotypic diversity of *P*. *infestans* in Northern China. Subsequently, a population analysis of *P*. *infestans* inferred from mtDNA haplotypes and restriction fragment length polymorphism (RFLP) data suggested multiple migrations of this pathogen into China (Guo et al., 2010). In addition, Tian et al. (2016) investigated the genetic structure of *P*. *infestans* populations in northwestern China using SSR markers and found that migration and selection might be important in shaping the genetic structure and diversity of regional populations. However, the population structure of *P*. *infestans* and the migration dynamics of this pathogen in China remain poorly understood.

This study analyzed the concatenated mtDNA sequences of the partial *cytochrome c* oxidase subunit 1 (*cox1*), NADH dehydrogenase subunit 9 (*nad9*), NADH dehydrogenase subunit 4 (*nad4*), and ATP synthase subunit alpha (*atp1*) genes from 101 *P*. *infestans* isolates collected in China. The objectives of this study were to: (i) characterize the genetic structure of *P*. *infestans* in China; (ii) investigate the geographic and host-origin effects on the population structure; and (iii) determine gene flow among populations, particularly in terms of the source–sink dynamics of the populations.

## Materials and Methods

### Sampling *P. infestans* and DNA Extraction

**FIGURE 1:**
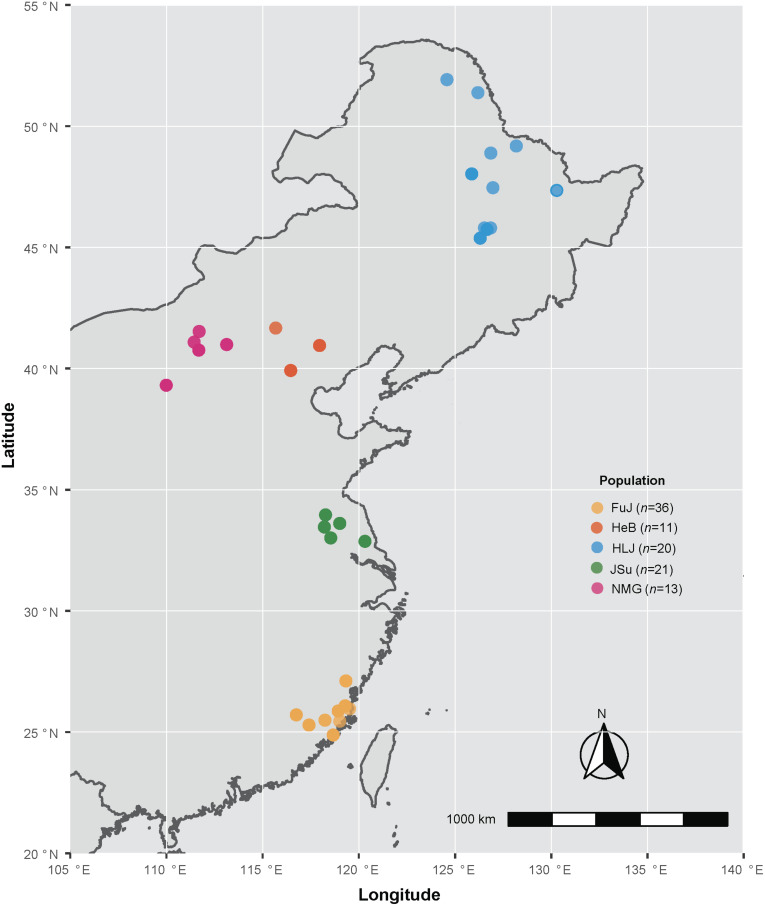
Map of the locations from which the *Phytophthora infestans* isolates were collected in this study. Colors indicate sampling locations, as shown in the key. Detailed information on the samples is given in Supplementary Table S1.

A total of 101 *P*. *infestans* infected potato (*Solanum tuberosum*) and tomato (*S. lycopersicum*) leaves, stems and tubers exhibiting typical late blight symptoms were collected from 34 sampling sites in Fujian, Hebei, Beijing, Heilongjiang, Inner Mongolia, and Jiangsu, China (Supplementary Table S1 and Figure 1). These isolates were grouped into five populations based on their geographical origin: Fujian (FuJ, *n* = 36), Hebei and Beijing (HeB, *n* = 11), Heilongjiang (HLJ, *n* = 20), Jiangsu (JSu, *n* = 21), and Inner Mongolia (NMG, *n* = 13). The pure culture of each isolate was preserved on antibiotic-free rye agar slants covered with mineral oil (Sigma Diagnostics Inc, St Louis, MO, United States) at 15°C in the dark and transferred to new slants at 6-month intervals. *P*. *infestans* genomic DNA was extracted using a modified cetyl trimethylammounium bromide (CTAB) method, as described previously (Li et al., 2009; Chen et al., 2012). The genomic DNA was eluted with 200 mL ultrapure water and stored in TE (10 mM Tris-HCl, 0.1 mM EDTA, pH8.0) at –20°C.

### PCR Amplification and Sequencing of the mtDNA Genes

Supplementary Table S2 lists the PCR primers and cycling parameters. PCR amplification was performed in a total volume of 25 μL, comprising 5 μL of TransTaq reaction buffer (5×), 2 μL of dNTPs (2.5 mM each), 1 μL each of forward and reverse primers (10 μmol/L), 14.25 μL of ddH_2_O,0.25 μL of *TaKaRa Ex Taq* DNA polymerase with the proofreading activity (5 U/μL), and 1 μL of template cDNA. The PCR program comprised an initial denaturation at 95°C for 2 min, followed by 30 cycles of 90°C for 20 s, 57°C for 25 s (for *cox1*; 41°C for *nad9* and 63°C for *nad4* and *atp1*; Supplementary Table S2), 72°C for 5 min; and a final 5 min extension at 72°C. The amplification products were separated by electrophoresis on 1% agarose gels, visualized by UV transillumination, and cleaned using an EasyPure Quick Gel Extraction Kit (TransGen, Beijing, China). The amplicons were ligated to pMD18-T Simple Vector (Takara, Beijing, China) and sequenced by Sangon Biological (Shanghai, China) using an ABI3730 automated DNA sequencer (Applied Biosystems, Foster City, CA, United States). At least three positive clones from each transformation were sequenced to obtain a consensus sequence.

### Sequence Dataset

The resulting sequences were assembled using DNAMAN 6.0 (Lynnon, Quebec, Canada) and deposited in GenBank under accession numbers MN458052 to MN45815 for *cox1*, MN458254 to MN458354 for *nad9*, MN458153 to MN458253 for *nad4*, and MN457951 to MN458051 for *atp1*. The four gene sequences were aligned by codons using TranslatorX (Abascal et al., 2010). A recent study found evidence for intragenic recombination in an effector gene of *P*. infestans (Yang et al., 2018). To test recombination signals in our data set, we conducted recombination analysis using the RDP 4.95 software package (Martin et al., 2015). No recombination signals were identified; therefore, we combined the four gene sequences for subsequent analyses.

### Genetic Diversity and Population Differentiation

To assess how genetic diversity varied across geographical and host populations, we calculated the two summary statistics, nucleotide diversity (*π*) and haplotype diversity (*H*_d_), using DnaSP 5.10 (Librado and Rozas, 2009). The nucleotide diversity was also calculated according to a sliding-window (100 bp) analysis, with a 30 bp step size estimate the step-wise diversity across the sequence. A minimum spanning haplotype network was built using PopART (Leigh and Bryant, 2015).

Genetic differentiation among populations was evaluated using *K*_ST_, *Z*, and *S*_nn_ using DnaSP 5.10 (Hudson et al., 1992; Hudson, 2000; Librado and Rozas, 2009). The level of genetic differentiation among *P*. *infestans* was also quantified using *F*_ST_ (Weir and Cockerham, 1984). We calculated pairwise *F*_ST_ values using noncorrected pairwise differences and obtained *p*-values after a randomization test with 10,000 permutations using Arlequin 3.5 (Excoffier and Lischer, 2010). Default values were used for the remaining parameters. The degree of differentiation (given by the *F*_ST_ values) was classified as low (<0.05), moderate (0.05–0.15), large (0.15–0.25); or great (> 0.25) (Balloux and Lugon-Moulin, 2002). We also applied discriminant analysis of principal components (DAPC), a multivariate method designed to identify clusters of genetically related individuals, to allow investigation of the genetic structure of *P*. *infestans* populations (Jombart et al., 2010). This method has the advantage of not assuming panmixia. We performed the DAPC analysis based on pre-defined groups, using the *adegenet* package (Jombart, 2008) implemented in *R* software (ver. 3.5.1; R Development Core Team, Vienna, Austria).

To test the effects of geography and host on the genetic diversity of *P*. *infestans*, we conducted analysis of molecular variance (AMOVA) using Arlequin 3.5 (Excoffier and Lischer, 2010). The statistical significance of the *φ*-statistics was tested based on 1,023 permutations (default). To determine the potential geographic and host-origin effects on *P*. *infestans* populations, the isolates were divided into five populations based on geographic origin, as described above. They were also divided into two populations based on host origin, i.e., *S*. *tuberosum* (potato, *n* = 70) and *S*. *lycopersicum* (tomato, *n* = 31). Pairwise population differentiation was also estimated based on *G*_ST_ using the *PopGenome* 2.16 R package (Pfeifer et al., 2014).

### Phylogeographic Analysis of *P*. *infestans*

To explore the spatial diffusion patterns of *P*. *infestans* across regions in China, we used Bayesian stochastic search variable selection (Lemey et al., 2009) to reconstruct the spatial pathway of this pathogen in BEAST 1.10.4 (Suchard et al., 2018). The five geographic locations in China were coded as discrete states. Before the analysis, we performed a date-randomization test (Ramsden et al., 2008) to analyze the temporal signal in the dataset using the *TipDatingBeast* package (Rieux and Khatchikian, 2017). However, we found no evidence of temporal structure in our dataset, so we applied a previous informative prior distribution of 1.5 × 10^–6^ to 3.3 × 10^–6^ substitutions/site/year on the clock rate (Yoshida et al., 2013). The best nucleotide substitution model, *GTR+F+I*, was identified by ModelFinder (Kalyaanamoorthy et al., 2017) with the Bayesian information criterion and used for analyzing the combined mtDNA gene sequences.

We calculated the marginal likelihoods using path sampling (Baele et al., 2012) and analyzed them in terms of Bayesian skyline, constant-size coalescent, and exponential-growth coalescent tree priors, as well as the strict clock and uncorrelated lognormal relaxed clock (Drummond et al., 2006). An exponential growth tree prior and uncorrelated lognormal relaxed clock gave the best fit to the data (Supplementary Table S3). The collection dates for all *P*. *infestans* samples were used as calibration points. Two independent Markov chain Monte Carlo (MCMC) runs were performed to estimate the posterior distributions of the parameters, with samples taken every 10,000 steps over 50,000,000 steps. After discarding the first 10% of the samples as burn-in, all parameters showed sufficient sampling, as confirmed by Tracer 1.7 (Rambaut et al., 2018), which showed that the effective sample size was above 200.

### Population Historic Dynamics of *P*. *infestans*

As neutrality indices, Tajima’s *D* and Fu’s *F*_S_ statistics were calculated using Arlequin 3.5 (Excoffier and Lischer, 2010). Tajima’s *D* test is based on comparison of the estimated number of segregating sites with a mean pairwise difference among sequences (Tajima, 1989). A negative Tajima’s *D* indicates bias toward rare alleles, which is indicative of recent population expansion. Fu’s *F*_S_ statistic is based on the allele or haplotype distribution and usually has a negative value, which is also indicative of a recent population expansion (Fu, 1997). We also used the exponential growth model implemented in BEAST to analyze the population demographic history of *P*. *infestans*.

## Results

### Genetic Diversity of *P*. *infestans* and Haplotype Network

We obtained 1,968 bp of mitochondrial genes in *P*. *infestans* in China, including *cox1* (735 bp), *nad9* (348 bp), *nad4* (396 bp), and *atp1* (489 bp). No indels were detected in our dataset. In the entire mtDNA, 141 polymorphic sites were identified, representing 7.16% of all sites analyzed, including 100 single variable sites and 41 parsimony-informative sites. The transition/transversion ratio (R) was 0.702, indicating a strong transversion bias during the mitochondrial evolution of *P*. *infestans*.

In total, 62 haplotypes were identified from the 101 *P*. *infestans* isolates studied, with a haplotype diversity of 0.920 (Table 1). The overall nucleotide sequence diversity of these isolates was 0.0023 (Table 1). When the *P*. *infestans* isolates were categorized according to geographic origin, the highest nucleotide diversity (0.00920 ± 0.00025) was observed in the FuJ population and the lowest (0.00153 ± 0.00033) was found in the HLJ population. Greater variation in the concatenated mtDNA sequences was observed in *P*. *infestans* isolates isolated from potato compared with those isolated from tomato, when the isolates were categorized according to host origin (Table 1).

**TABLE 1:** Genetic diversity and neutrality tests for *P. infestans* population in China.

Population	*n*	*h*	*H*_d_ (S.D.)	*π* (S.D.)	Tajima’s *D*	Fu’s *F*_S_
**REGION**						
FuJ	36	15	0.695 ± 0.0870	0.00920 ± 0.00025	−2.334**	−10.124***
HeB	11	8	0.927 ± −0.066	0.00309 ± 0.00010	−1.873*	−1.221^ns^
HLJ	20	14	0.889 ± 0.068	0.00153 ± 0.00033	−2.333***	−9.392***
JSu	21	18	0.971 ± 0.030	0.00308 ± 0.00043	−2.177**	−10.024***
NMG	13	12	0.987 ± 0.035	0.00433 ± 0.00074	−1.785*	−4.653*
Combined	101	62	0.920 ± 0.021	0.00230 ± 0.00025	−2.103**	−7.083*
**HOST**						
Potato	70	51	0.959 ± 0.017	0.00292 ± 0.00032	−2.684***	−25.350***
Tomato	31	12	0.360 ± 0.102	0.00058 ± 0.00017	−2.237**	−9.333***
Combined	101	62	0.920 ± 0.021	0.00230 ± 0.00025	−2.460***	−17.342***

n, sample size; H_d_, haplotype diversity; π, nucleotide diversity; **0.01 < p < 0.05, **0.001 < p < 0.01, ***p < 0.001; ns, not significant.

**FIGURE 2:**
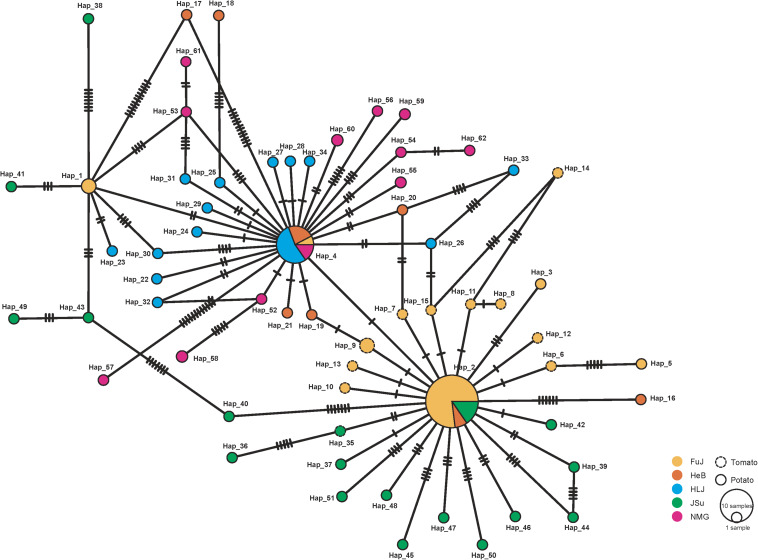
Minimum spanning haplotype network inferred from the concatenated mitochondrial DNA (mtDNA) sequences of *Phytophthora infestans* in China. Sequences from different sampling locations are indicated in unique colors and the sizes of the circles represent the haplotype frequencies in populations.

**FIGURE 3:**
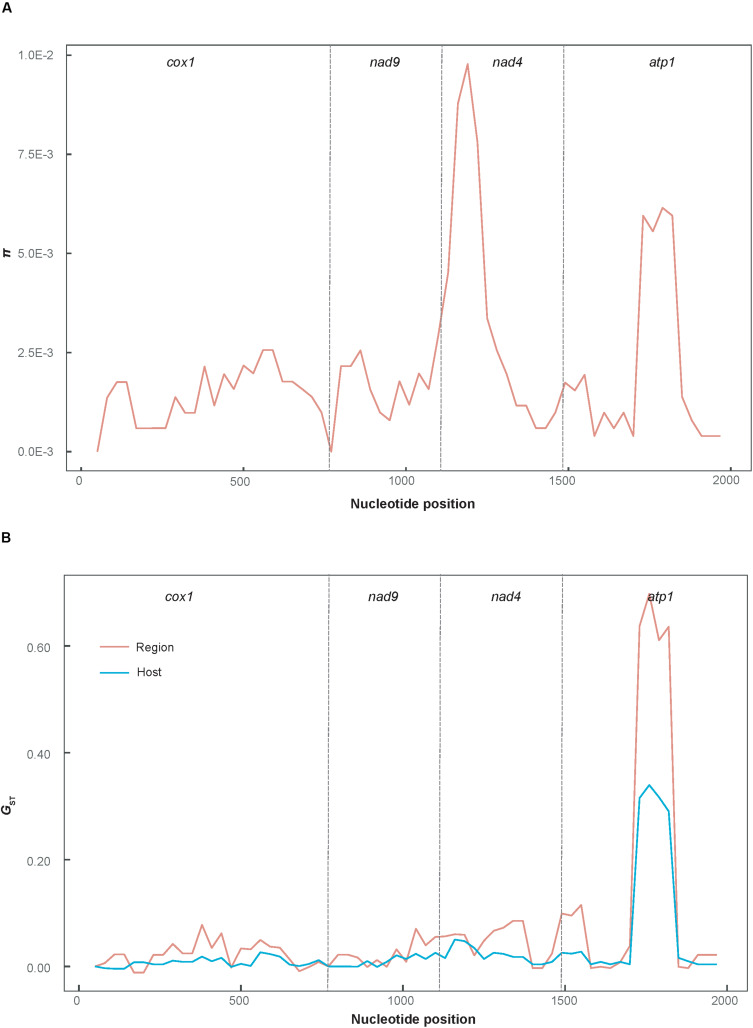
Sliding-window plot of nucleotide diversity **(A)** and population differentiation **(B)** for the concatenated mtDNA sequences. *π* was calculated using DnaSP 5.10 and *G*_ST_ was plotted using the *PopGenome* 2.16 *R* package (sliding-window analysis; length, 100 bp; step size, 30 bp).

Figure 2 shows the minimum spanning haplotype network of the *P*. *infestans* population. There were 58 low-frequency haplotypes, each found in a single *P*. *infestans* isolate. Four haplotypes were shared by two or more isolates. Hap_2 and Hap_4 were commonly shared haplotypes, being present in 26 and 13 isolates, respectively. Of all the identified haplotypes, 11 private haplotypes were found in the isolates from tomato, of which 10 were from the FuJ population and one was from the JSu population. The population network showed low levels of sequence divergence and a high frequency of unique mutations, which is a signature of rapid population expansion. The step-wise diversity across the sequence indicated two highly variable regions at sites 1111–1270 and 1681–1870 (Figure 3), which were located within the *nad4* and *atp1* genes, respectively.

### Differentiation Between *P*. *infestans* Populations

With the exception of the comparison between HLJ and HeB, three independent tests of population differentiation for all populations, grouped by geography or host-species, were significant (Table 2); the *F*_ST_ values ranged from 0.028 to 0.289. The highest *F*_ST_ value was between the HLJ and FuJ populations and the lowest was between NMG and HeB (Table 2). More than half of the 10 *F*_ST_ values were higher than 0.05, suggesting a degree of spatial structure of the *P*. *infestans* populations in China. Our investigation of *F*_ST_ also showed significant genetic differentiation between *P*. *infestans* from tomato versus potato.

**TABLE 2:** Genetic differentiation between *Phytophthora infestans* populations.

Population	*K*_ST_	*K*_S_	Z*	*S*_nn_	*F*_ST_
**REGION**					
FuJ vs. HeB	0.050***	2.786	5.984***	0.801***	0.168***
FuJ vs. HLJ	0.158***	2.075	6.163***	0.917***	0.289**
FuJ vs. JSU	0.014**	3.360	6.354***	0.630***	0.041***
FuJ vs. NMG	0.106***	3.389	5.595***	0.918***	0.263***
HeB vs. HLJ	0.004^ns^	3.795	5.184^ns^	0.594^ns^	0.025^ns^
HeB vs. JSu	0.046***	6.031	5.169*	0.740**	0.095***
HeB vs. NMG	0.0185**	7.105	4.620^ns^	0.641*	0.0283*
HLJ vs. JSu	0.109***	4.446	5.499***	1.000***	0.194***
HLJ vs. NMG	0.0148**	4.641	5.225*	0.669**	0.044**
JSu vs. NMG	0.077***	6.857	5.190***	1.000***	0.149***
**HOST**					
Potato vs. tomato	0.043***	4.229	7.394***	0.757***	0.087***

Significance thresholds: *0.01<p < 0.05; **0.001<p < 0.01; ***p < 0.001; ns, not significant.

**FIGURE 4:**
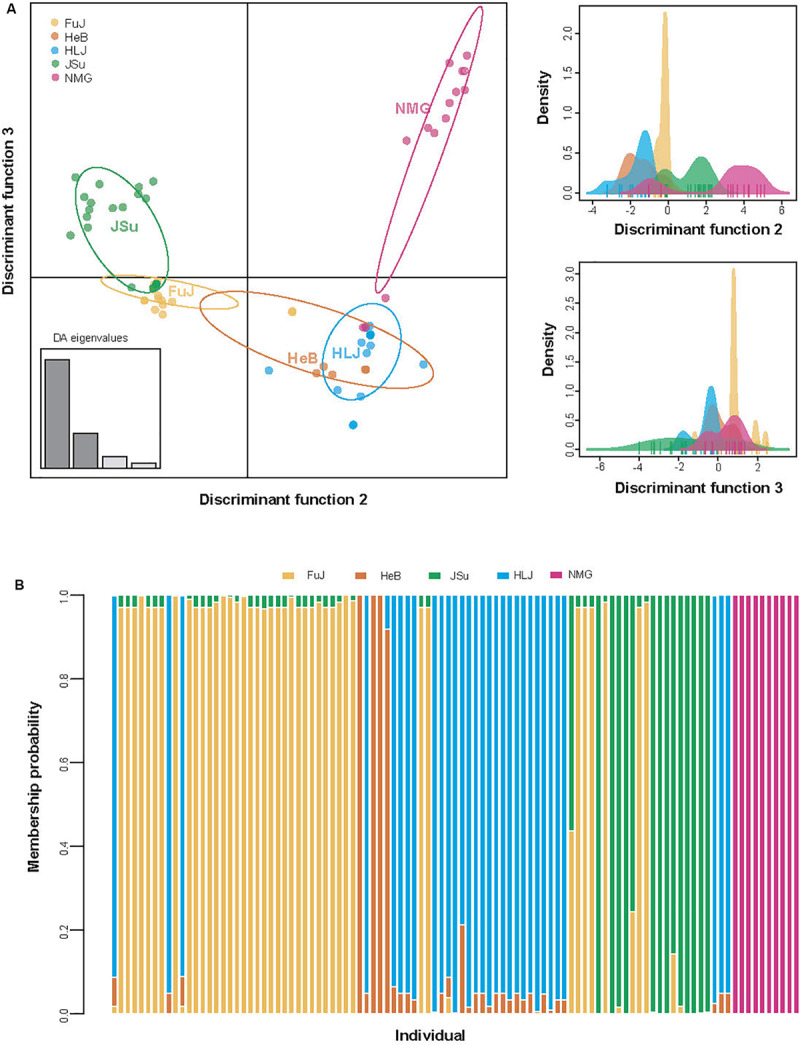
Population structure of *Phytophthora infestans* according to the discriminant analysis of principal components (DAPC). **(A)** Scatterplots for individual isolates. Unique colors indicate geographic regions, as shown in the insert. The *x*- and *y*-axes, respectively, indicate the second and third discriminant principal components, which best summarize the differences between clusters while neglecting within-cluster variation. The discriminant functions used for separating the clusters are shown in the right panels. **(B)** Membership probability of individual isolates.

The DAPC analysis revealed patterns of genetic differentiation that were similar to those of the *F*_ST_ analysis. DAPC scatterplots also indicated that the NMG population was relatively distinct from the other populations along the second discriminant function axis, while the JSu population had a more subtle structural difference along the third discriminant function axis (Figure 4). This suggests that geography contributes to the differentiation of *P*. *infestans* populations.

AMOVA also revealed significant variation among geographic groups, accounting for 13.87% of the total variation in *P*. *infestans* (***Φ***_ST_ = 0.139, *p* < 0.001; Table 3). When host species was used a grouping factor, similar results were observed, i.e., there was significant population differentiation among groups (***Φ***_ST_ = 0.087, *p* < 0.001), accounting for nearly 8.66% of the total variation in *P*. *infestans* (Table 3). These results suggest that the effect of geography on the genetic variance of *P*. *infestans* is greater than that of host species, although both contributed to the genetic variance in *P*. *infestans*.

**TABLE 3:** Analysis of molecular variance for *Phytophthora infestans* population in China.

Group factors	Source of variation	d.f.	Sum of squares	Variance components	Percentage of variation	Fixation index
Region	Among groups	4	32.228	0.317 Va	13.87	***Φ***_ST_ = 0.139***
	Within groups	96	188.722	1.966 Vb	86.13	
	Total	100	220.950	2.283		
Host	Among groups	1	10.768	0.201	8.66	***Φ***_ST_ = 0.087***
	Within groups	99	210.182	2.123	91.34	
	Total	100	220.950	2.324		

d.f., degree of freedom; significance thresholds: ***p < 0.001.

The results of sliding-window analysis of the pairwise *G*_ST_ values of population differentiation are plotted in Figure 3B. The pairwise *G*_ST_ values estimated based on the geographic groupings were slightly higher than those based on the host species groupings, across the sequence regions located in the *cox1*, *nad9*, and *nad4* genes. However, the former values were significantly higher than the latter in the highly variable region in the *atp1* gene (alignment sites 1681–1870).

### The Dispersal Pattern of *P*. *infestans* Populations in China

**FIGURE 5:**
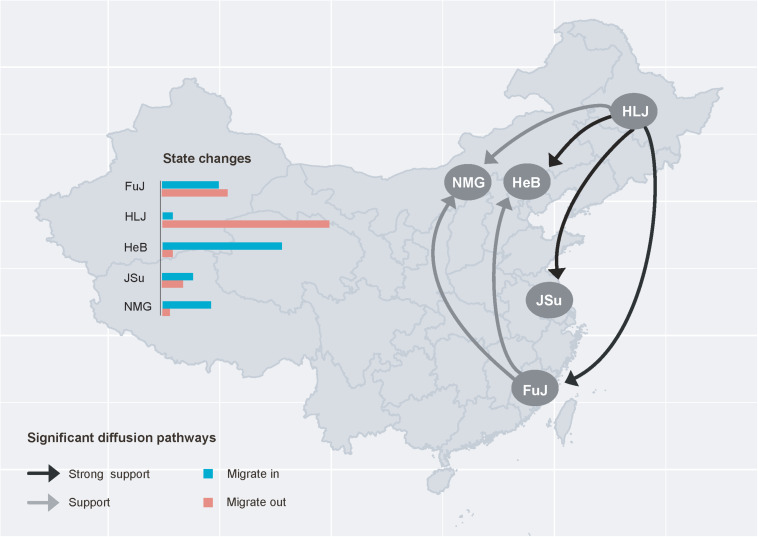
Spatial diffusion pathway of *Phytophthora infestans* inferred from 101 isolates collected from five geographic regions. Histograms of the total number of location-state transitions are shown in the insert panel. FuJ, Fujian; HLJ, Heilongjiang; HeB, Hebei; JSu, Jiangsu; NMG, Inner Mongolia.

All *F*_ST_ values were lower than 0.33 (Table 2), indicating a degree of gene flow between these populations. To gain insight into the circulation of *P*. *infestans* across China, we reconstructed the migration pathway of this pathogen. Our Bayesian phylogeographic analysis suggested that there were six major migration events in the diffusion process of *P*. *infestans*, with mean migration rates ranging from 0.922 to 1.323; four of the migrations were from HLJ to HeB, NMG, JSu, and FuJ, and the remaining two were from FuJ to HeB and NMG (Figure 5 and Supplementary Table S4). The observed state changes (MCMC jumps) also suggest that HLJ has acted as the seeding population for *P*. *infestans* in China (Figure 5). The Markov rewards for FuJ (14,748; 95% credibility interval, 7,756–50,530) were higher than those for other regions, suggesting that Fujian played a major role in the evolution and persistence of *P*. *infestans*.

### Demographic History of the *P*. *infestans* Population in China

**FIGURE 6:**
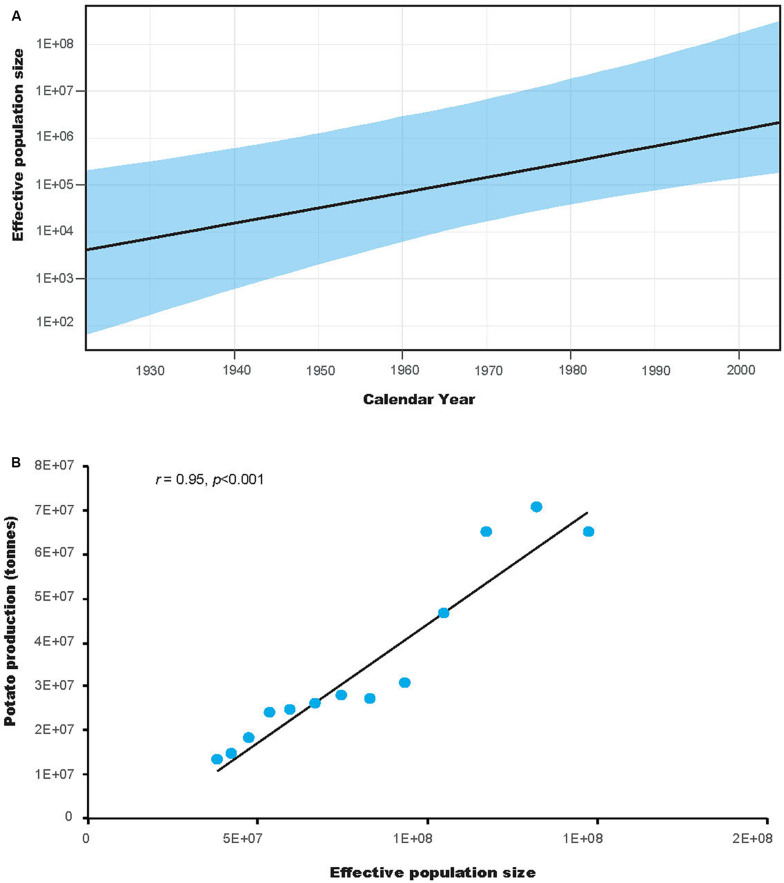
The demographic history of *Phytophthora infestans* and its correlation with the production of potato in China **(A)** Population dynamics of *P. infestans*. The *x*-axis units are years, and the *y*-axis is the effective population size, which provides a measure of relative genetic diversity. The areas in light blue are within the 95% highest posterior density interval. **(B)** Correlation between the effective population size and potato production in China. The *y*-axis units are tons.

Tajima’s *D* test was significantly negative for all populations. Fu’s *F*_S_ test was also significantly negative for all populations, except the HeB population (Table 1). The negative values of both neutrality tests indicate an excess of rare mutations, suggesting recent population expansion. This is also indicated by the results of the coalescence-based demographic analysis, which showed that *P*. *infestans* had experienced substantial population expansion until the last sampling year (Figure 6A). Interestingly, the demographic expansion of *P. infestans* correlates with the increase of potato production in China over the past decades (*r* = 0.95, *DF* = 23, *p* = 0.000, Figure 6B).

## Discussion

We obtained new sequence data for four mtDNA genes from 101 isolates of *P*. *infestans* in China. Using these data, we first investigated the genetic diversity of this pathogen. High haplotype diversity and low *π* was found. This differs from a recent study, which reported high genetic diversity in the effector gene *Avr3a* of 96 *P*. *infestans* isolates collected from six potato-planting regions in China (Yang et al., 2018). In addition to genetic markers, the major difference between the two studies was that only isolates with distinct genotypes were chosen in the previous, but not the current study (Yang et al., 2018). However, high genetic variation was also found in the mtDNA genes in the current study. In total, 62 nucleotide haplotypes (Table 1) and 70 isoforms (data not shown) were detected in the 101 sequences. The genetic variation is comparable to that of *P*. *infestans* nuclear genes (Yang et al., 2018).

Despite the high haplotype diversity of the *P*. *infestans* population, the low *π* suggests only small differences between haplotypes. This was also observed in the minimum spanning haplotype network (Figure 2). The high haplotype and low *π* observed in this study indicates rapid population expansion from a small effective population size (Grant and Bowen, 1998). Based on FAO data^2^, the production of potato in China increased from ∼12.91 million tons in 1961 to 6.45 million tons in 2006. This coincidence suggested a link between potato cultivation area and the population size of *P*. *infestans*. Indeed, our results supported this with a positive correlation between the demographic history and the production of potato in China (Figure 6B). Further studies aiming to understand how human activities influences the demographic expansion of the *P*. *infestans* population will be interesting.

Among the five geographic populations of *P*. *infestans*, the FuJ population had the highest genetic diversity. One potential reason for this is that the FuJ population may have acted as an important hub for the spread of *P*. *infestans* in China, because Fujian Province, a winter potato-cropping zone, imports seed tubers extensively from other cropping zones. This is also supported by the results regarding the MCMC jumps (Figure 5). Comparatively, the genetic variation was higher in *P*. *infestans* from potato than from tomato (Table 1). This might be due to the larger sample size of potato sequences compared to tomato sequences (70 vs. 31 isolates). When we recomputed the haplotype diversity after standardizing sample sizes with the smaller population (tomato in this case) using a bootstrapping approach, the difference in genetic variation between the two populations disappeared (*p*> 0.5, data not shown).

Current studies usually use the relatively short, reasonably variable gene fragments flanked by highly conserved sequences as mtDNA markers, such as the *cox1* gene, which was used for DNA barcoding to identify plants and fungi (Robba et al., 2006; Stockinger et al., 2010). For intraspecific studies, slow mtDNA evolution generally results in low levels of polymorphism in mtDNA genome. A similar observation has been made for the *eEF-1α* gene of *P*. *infestans* (Wang et al., 2020). The *atp1* gene is a rotary enzyme that exploits the proton gradient across the inner membrane, generated by the electron transport chain, to synthesize ATP (Song et al., 2018), however, it had the highest variability among the four mtDNA genes examined in this study, as indicated in Figure 3. This gene can distinguish closely related *P*. *infestans* populations between different geographic regions or host species; therefore, it could be an ideal genetic marker for population genetic analysis.

Our phylogeographic analysis revealed multiple north-to-south dispersal events in *P*. *infestans* in China (Figure 5 and Supplementary Table S4). This accords with the fact that seed potatoes are normally produced in northern China, suggesting that the pattern of *P*. *infestans* dispersal is associated with human-mediated activities. In China, the majority of potatoes in the winter-cropping and central double-cropping zones in southern China are intended for export, and not for planting, while seed potatoes are supplied from the northern single-cropping zone. Further studies to understand how human-mediated activities influence the migration of *P*. *infestans* should be informative.

## Conclusion

In conclusion, this study examined the population genetic structure and phylogeography of *P*. *infestans*. The results showed high haplotype diversity, but low nucleotide diversity, of *P*. *infestans* in China. The diversification patterns are likely shaped by gene flow. We also found multiple north-to-south migration pathways of *P*. *infestans* in China, suggesting that the movement of seed potato tubers through the country has played a major role in the dispersal of *P*. *infestans* in China. This information is potentially valuable for the development of more effective strategies to control this pathogen. Further analyses of larger datasets with *P*. *infestans* outside of China will lead to a more comprehensive view of the evolutionary history of this pathogen.

## Data Availability Statement

The datasets generated for this study can be found in the GenBank database under accession numbers MN458052 to MN45815 for *cox1*, MN458254 to MN458354 for *nad9*, MN458153 to MN458253 for *nad4*, and MN457951 to MN458051 for *atp1*.

## Author Contributions

QC and FG conceived and designed the experiments and analyzed the data. CC, FG, BL, and QW performed the experiments. QC and FG wrote the manuscript. All the authors reviewed the manuscript.

## Conflict of Interest

The authors declare that the research was conducted in the absence of any commercial or financial relationships that could be construed as a potential conflict of interest.

## Footnotes

1 http://www.fao.org/faostat

2 http://faostat.fao.org
