# Supplementary material for: The Gene Flow Direction of Geographically Distinct Phytophthora infestans Populations in China Corresponds With the Route of Seed Potato Exchange
Source: Front Microbiol. 2020 May 26;11:1077. doi: 10.3389/fmicb.2020.01077 (PMC7264822; doi:10.3389/fmicb.2020.01077)
Supplement: Supplementary file 1 [file Table_1.DOCX]

**Table S1 *Phytophthora infestans* isolates used in this study**

| **No** | **Strain** | **Province** | **Host** | **Year** | **Population** |
| --- | --- | --- | --- | --- | --- |
| 1 | CLP-2 | Fujian | *Solanum tuberosum* | 2001 | FuJ |
| 2 | PT-7 | Fujian | *Solanum tuberosum* | 2001 | FuJ |
| 3 | SC1-6 | Fujian | *Solanum lycopersicum* | 2001 | FuJ |
| 4 | SC1-9 | Fujian | *Solanum lycopersicum* | 2001 | FuJ |
| 5 | SC1-15 | Fujian | *Solanum lycopersicum* | 2001 | FuJ |
| 6 | SC1-16 | Fujian | *Solanum lycopersicum* | 2001 | FuJ |
| 7 | DH3-1 | Fujian | *Solanum tuberosum* | 2002 | FuJ |
| 8 | DH2-2 | Fujian | *Solanum tuberosum* | 2002 | FuJ |
| 9 | QZ 2-2 | Fujian | *Solanum tuberosum* | 2002 | FuJ |
| 10 | YT-2 | Fujian | *Solanum tuberosum* | 2002 | FuJ |
| 11 | YT-5 | Fujian | *Solanum tuberosum* | 2002 | FuJ |
| 12 | YT1-6 | Fujian | *Solanum tuberosum* | 2002 | FuJ |
| 13 | ZN-1 | Fujian | *Solanum tuberosum* | 2002 | FuJ |
| 14 | ZN-5 | Fujian | *Solanum tuberosum* | 2002 | FuJ |
| 15 | ZN-12 | Fujian | *Solanum tuberosum* | 2002 | FuJ |
| 16 | LC-a | Fujian | *Solanum lycopersicum* | 2004 | FuJ |
| 17 | LC-b | Fujian | *Solanum lycopersicum* | 2004 | FuJ |
| 18 | LC-51b | Fujian | *Solanum lycopersicum* | 2004 | FuJ |
| 19 | LC-2 | Fujian | *Solanum lycopersicum* | 2004 | FuJ |
| 20 | LC-4 | Fujian | *Solanum lycopersicum* | 2004 | FuJ |
| 21 | 2-2 | Fujian | *Solanum lycopersicum* | 2005 | FuJ |
| 22 | 2-2_FJ | Fujian | *Solanum lycopersicum* | 2005 | FuJ |
| 23 | ZP-2 | Fujian | *Solanum lycopersicum* | 2005 | FuJ |
| 24 | ZP3-4-2 | Fujian | *Solanum lycopersicum* | 2005 | FuJ |
| 25 | ZP3-5 | Fujian | *Solanum lycopersicum* | 2005 | FuJ |
| 26 | YF5-1 | Fujian | *Solanum lycopersicum* | 2006 | FuJ |
| 27 | YF06-2 | Fujian | *Solanum lycopersicum* | 2006 | FuJ |
| 28 | YF06-10 | Fujian | *Solanum lycopersicum* | 2006 | FuJ |
| 29 | YF06-12 | Fujian | *Solanum lycopersicum* | 2006 | FuJ |
| 30 | LC06-1 | Fujian | *Solanum lycopersicum* | 2006 | FuJ |
| 31 | LC06-3 | Fujian | *Solanum lycopersicum* | 2006 | FuJ |
| 32 | LC06-5 | Fujian | *Solanum lycopersicum* | 2006 | FuJ |
| 33 | LCXP06-6 | Fujian | *Solanum lycopersicum* | 2006 | FuJ |
| 34 | LCXP06-13 | Fujian | *Solanum lycopersicum* | 2006 | FuJ |
| 35 | LCXP06-18 | Fujian | *Solanum lycopersicum* | 2006 | FuJ |
| 36 | LCXP06-26 | Fujian | *Solanum lycopersicum* | 2006 | FuJ |
| 37 | 91001 | Beijing | *Solanum lycopersicum* | 2004 | HeB |
| 38 | 89148-07 | Beijing | *Solanum lycopersicum* | 2004 | HeB |
| 39 | WCD-1 | Hebei | *Solanum tuberosum* | 2004 | HeB |
| 40 | 614 | Hebei | *Solanum tuberosum* | 2006 | HeB |
| 41 | W2-6-1 | Hebei | *Solanum tuberosum* | 2006 | HeB |
| 42 | W3-1-2 | Hebei | *Solanum tuberosum* | 2006 | HeB |
| 43 | W3-6-1 | Hebei | *Solanum tuberosum* | 2006 | HeB |
| 44 | W5-2-1 | Hebei | *Solanum tuberosum* | 2006 | HeB |
| 45 | W6-3-1 | Hebei | *Solanum tuberosum* | 2006 | HeB |
| 46 | W7-6-3 | Hebei | *Solanum tuberosum* | 2006 | HeB |
| 47 | W9-8-2 | Hebei | *Solanum tuberosum* | 2006 | HeB |
| 48 | 220 | Heilongjiang | *Solanum tuberosum* | 2001 | HLJ |
| 49 | DA-6 | Heilongjiang | *Solanum tuberosum* | 2004 | HLJ |
| 50 | W3 | Heilongjiang | *Solanum tuberosum* | 2004 | HLJ |
| 51 | W4 | Heilongjiang | *Solanum tuberosum* | 2004 | HLJ |
| 52 | W7 | Heilongjiang | *Solanum tuberosum* | 2004 | HLJ |
| 53 | LM-4 | Heilongjiang | *Solanum tuberosum* | 2004 | HLJ |
| 54 | HL-4 | Heilongjiang | *Solanum tuberosum* | 2004 | HLJ |
| 55 | DA-3 | Heilongjiang | *Solanum tuberosum* | 2004 | HLJ |
| 56 | KS-1 | Heilongjiang | *Solanum tuberosum* | 2004 | HLJ |
| 57 | W18 | Heilongjiang | *Solanum tuberosum* | 2005 | HLJ |
| 58 | W21 | Heilongjiang | *Solanum tuberosum* | 2005 | HLJ |
| 59 | W24 | Heilongjiang | *Solanum tuberosum* | 2005 | HLJ |
| 60 | W10 | Heilongjiang | *Solanum tuberosum* | 2005 | HLJ |
| 61 | W34 | Heilongjiang | *Solanum tuberosum* | 2005 | HLJ |
| 62 | W17 | Heilongjiang | *Solanum tuberosum* | 2005 | HLJ |
| 63 | HK-1 | Heilongjiang | *Solanum tuberosum* | 2006 | HLJ |
| 64 | HK-4 | Heilongjiang | *Solanum tuberosum* | 2006 | HLJ |
| 65 | HK-5 | Heilongjiang | *Solanum tuberosum* | 2006 | HLJ |
| 66 | HK-10 | Heilongjiang | *Solanum tuberosum* | 2006 | HLJ |
| 67 | HK-11 | Heilongjiang | *Solanum tuberosum* | 2006 | HLJ |
| 68 | 204 | Inner Mongolia | *Solanum tuberosum* | 1998 | NMG |
| 69 | 222 | Inner Mongolia | *Solanum tuberosum* | 1998 | NMG |
| 70 | NM324 | Inner Mongolia | *Solanum tuberosum* | 2000 | NMG |
| 71 | 272 | Inner Mongolia | *Solanum tuberosum* | 2002 | NMG |
| 72 | NM256 | Inner Mongolia | *Solanum tuberosum* | 2002 | NMG |
| 73 | NM345 | Inner Mongolia | *Solanum tuberosum* | 2002 | NMG |
| 74 | 276 | Inner Mongolia | *Solanum tuberosum* | 2003 | NMG |
| 75 | 285 | Inner Mongolia | *Solanum tuberosum* | 2003 | NMG |
| 76 | NM332 | Inner Mongolia | *Solanum tuberosum* | 2003 | NMG |
| 77 | NM307 | Inner Mongolia | *Solanum tuberosum* | 2003 | NMG |
| 78 | 290 | Inner Mongolia | *Solanum tuberosum* | 2004 | NMG |
| 79 | NM378 | Inner Mongolia | *Solanum tuberosum* | 2004 | NMG |
| 80 | NM312 | Inner Mongolia | *Solanum tuberosum* | 2004 | NMG |
| 81 | JS-SQ1 | Jiangsu | *Solanum tuberosum* | 2001 | JSu |
| 82 | JS-HA1 | Jiangsu | *Solanum tuberosum* | 2001 | JSu |
| 83 | JS-XY1 | Jiangsu | *Solanum tuberosum* | 2002 | JSu |
| 84 | JS-XY2 | Jiangsu | *Solanum tuberosum* | 2002 | JSu |
| 85 | JS-DT3 | Jiangsu | *Solanum tuberosum* | 2003 | JSu |
| 86 | JS-DT4 | Jiangsu | *Solanum tuberosum* | 2003 | JSu |
| 87 | JS-XY3 | Jiangsu | *Solanum tuberosum* | 2003 | JSu |
| 88 | JS-XY4 | Jiangsu | *Solanum tuberosum* | 2003 | JSu |
| 89 | JS-SQ2 | Jiangsu | *Solanum tuberosum* | 2003 | JSu |
| 90 | JS-SQ4 | Jiangsu | *Solanum tuberosum* | 2003 | JSu |
| 91 | JS-HA2 | Jiangsu | *Solanum tuberosum* | 2003 | JSu |
| 92 | JS-HA3 | Jiangsu | *Solanum tuberosum* | 2003 | JSu |
| 93 | JS-DT1 | Jiangsu | *Solanum tuberosum* | 2004 | JSu |
| 94 | JS-DT2 | Jiangsu | *Solanum tuberosum* | 2004 | JSu |
| 95 | JS-HA4 | Jiangsu | *Solanum tuberosum* | 2004 | JSu |
| 96 | JS-HA5 | Jiangsu | *Solanum tuberosum* | 2004 | JSu |
| 97 | P.six | Jiangsu | *Solanum lycopersicum* | 2005 | JSu |
| 98 | P.seven | Jiangsu | *Solanum lycopersicum* | 2005 | JSu |
| 99 | P.nine | Jiangsu | *Solanum lycopersicum* | 2005 | JSu |
| 100 | P.ten | Jiangsu | *Solanum lycopersicum* | 2005 | JSu |
| 101 | JS-SQ3 | Jiangsu | *Solanum tuberosum* | 2005 | JSu |
