# Supplementary material for: The Gene Flow Direction of Geographically Distinct Phytophthora infestans Populations in China Corresponds With the Route of Seed Potato Exchange
Source: Front Microbiol. 2020 May 26;11:1077. doi: 10.3389/fmicb.2020.01077 (PMC7264822; doi:10.3389/fmicb.2020.01077)
Supplement: Supplementary file 2 [file Table_2.DOCX]

**Table S2.** Primers used to amplify *cox1*, *nad9, nad4* and *atp1* genes of *Phytophthora. infestans*

| **Region** | **Name** | **Sequence (5 🡪3´)** | ***T*_m_ （**℃**）** | **Expected size (bp)** |
| --- | --- | --- | --- | --- |
| *cox1* | F4 | 5'-TGGTCATCCAGAGGTTTATGTT-3' | 57 | 964 |
|  | R4 | 5'-CCGATACCGATACCAGCACCAA-3' |  |  |
| *nad9* | NADF1 | 5'-TATAATTTATTAAGTATACGTTT-3' | 41 | 783 |
|  | NADR1 | 5'-CATCATTAAAATAAAAATAATTA-3' |  |  |
| *nad4* and *atp1* | F2 | 5'-TTCCCTTTGTCCTCTACCGAT-3' | 63 | 1070 |
|  | R2 | 5'-TTACGGCGGTTTAGCACATACA-3' |  |  |
