# Supplementary material for: The Gene Flow Direction of Geographically Distinct Phytophthora infestans Populations in China Corresponds With the Route of Seed Potato Exchange
Source: Front Microbiol. 2020 May 26;11:1077. doi: 10.3389/fmicb.2020.01077 (PMC7264822; doi:10.3389/fmicb.2020.01077)
Supplement: Supplementary file 3 [file Table_3.DOCX]

**Table S3** Marginal likelihoods of different combinations of clock model and tree prior

| **No.** | **Site model** | **Clock model** | **Tree prior** | **Path sampling** | **Step sampling** |
| --- | --- | --- | --- | --- | --- |
| 1 | *GTR+F+I* | Strict | Bayesian skyline | - 4451.343 | - 4452.863 |
| 2 | *GTR+F+I* | Strict | Constant size | - 4474.751 | - 4472.412 |
| 3 | *GTR+F+I* | Strict | Exponential growth | - 4453.805 | - 4451.241 |
| 4 | *GTR+F+I* | UCLN | Bayesian skyline | - 4430.903 | - 4432.593 |
| 5 | *GTR+F+I* | UCLN | Constant size | - 4459.355 | - 4457.223 |
| **6** | ***GTR+F+I*** | **UCLN** | **Exponential growth** | **- 4429.682** | **- 4427.396** |

UCLN, Uncorrelated lognormal relaxed clock

The best-fitting tree prior and molecular clock model are indicated in bold font.
