# Supplementary material for: The Gene Flow Direction of Geographically Distinct Phytophthora infestans Populations in China Corresponds With the Route of Seed Potato Exchange
Source: Front Microbiol. 2020 May 26;11:1077. doi: 10.3389/fmicb.2020.01077 (PMC7264822; doi:10.3389/fmicb.2020.01077)
Supplement: Supplementary file 4 [file Table_4.DOCX]

**Table S4** Statistically supported migration rates of *Phytophthora infestans* estimated from the combined mtDNA sequence

| **From** | **To** | **Mean migration rate ^a^** | **Indicator ^b^** | **Bayes factor ^c^** |
| --- | --- | --- | --- | --- |
| FuJ | HeB | 1.085 | 0.725 | 8.586 |
| FuJ | NMG | 0.922 | 0.542 | 3.854 |
| HLJ | HeB | 1.931 | 0.903 | 30.443 |
| HLJ | NMG | 1.044 | 0.658 | 6.273 |
| HLJ | JSu | 0.972 | 0.843 | 17.515 |
| HLJ | FuJ | 1.323 | 0.849 | 18.404 |

^a^ Unit: migration events per lineage per year

^b^ Posterior probability of observing a non-zero migration rate in the sampled trees

^c^ Only statistically supported migrations with indicator values >0. 50 and BF >3 are shown
